# Identifying stress responsive genes using overlapping communities in co-expression networks

**DOI:** 10.1186/s12859-021-04462-4

**Published:** 2021-11-07

**Authors:** Camila Riccio-Rengifo, Jorge Finke, Camilo Rocha

**Affiliations:** 1 Department of Natural Sciences and Mathematics, Pontificia Universidad Javeriana, Cali, Colombia; 2 Department of Electronics and Computer Science, Pontificia Universidad Javeriana, Cali, Colombia

**Keywords:** Stress-responsive genes, Co-expression network, Overlapping communities, Phenotypic traits, LASSO, Salinity, Rice, Oryza sativa

## Abstract

**Background:**

This paper proposes a workflow to identify genes that respond to specific treatments in plants. The workflow takes as input the RNA sequencing read counts and phenotypical data of different genotypes, measured under control and treatment conditions. It outputs a reduced group of genes marked as relevant for treatment response. Technically, the proposed approach is both a generalization and an extension of WGCNA. It aims to identify specific modules of overlapping communities underlying the co-expression network of genes. Module detection is achieved by using Hierarchical Link Clustering. The overlapping nature of the systems’ regulatory domains that generate co-expression can be identified by such modules. LASSO regression is employed to analyze phenotypic responses of modules to treatment.

**Results:**

The workflow is applied to rice (*Oryza sativa*), a major food source known to be highly sensitive to salt stress. The workflow identifies 19 rice genes that seem relevant in the response to salt stress. They are distributed across 6 modules: 3 modules, each grouping together 3 genes, are associated to shoot K content; 2 modules of 3 genes are associated to shoot biomass; and 1 module of 4 genes is associated to root biomass. These genes represent target genes for the improvement of salinity tolerance in rice.

**Conclusions:**

A more effective framework to reduce the search-space for target genes that respond to a specific treatment is introduced. It facilitates experimental validation by restraining efforts to a smaller subset of genes of high potential relevance.

## Introduction

Stresses are key factors that influence plant development, often associated to extensive losses in agricultural production [1, 2]. Soil salinity is one of the most devastating abiotic stresses. According to [2], soil salinity contributes to a significant reduction in areas of cultivable land and crop quality. The study estimates that 20% of the total cultivated land worldwide and 33% of the total irrigated agricultural land is affected by high salinity. By the end of 2050, areas of high salinity are expected to reach 50% of the cultivated land [2].

Salinity tolerance and susceptibility are the result of elaborated interactions between morphological, physiological, and biochemical processes. They are regulated by multiple genes in various parts of the plant genome [3]. Consequently, identifying groups of responsive genes is an important step for improving crop varieties in terms of salinity tolerance. This paper proposes a workflow to identify stress responsive genes associated with a complex quantitative trait.

To discover the genes associated with a phenotypic response to treatment, the workflow takes as input the gene expression profiles of the target organism. Specifically, it takes the RNA sequencing read counts (measured under control and treatment conditions) of at least two biological replicates per genotype. It also receives phenotypic data in the form of observable traits, measured for each genotype under the two conditions. The output of the workflow is a set of genes that are characterized as potentially relevant to treatment.

Broadly speaking, the workflow provides a framework that yields insight into the possible behavior of specific genes and the role they play in functional pathways in response to treatment. It takes advantage of the current availability of high-throughput technologies, which enable the access to transcriptomic data of organisms under different conditions and a better understanding of their reaction under different environmental stimuli.

The proposed approach is both a generalization and an extension of the Weighted Gene Co-expression Network Analysis (WGCNA) [4, 5]. Like WGCNA, the general idea behind the proposed approach is to identify, after a sequence of normalization and filtering steps, specific modules of overlapping communities underlying the co-expression network of genes. The proposed approach is considered a *generalization* of WGCNA because module detection recognizes overlapping communities using the Hierarchical Link Clustering (HLC) [6] algorithm. Conceptually, the generalization adds the overlapping nature of the regulatory domains of the systems that generate the co-expression network [7]. The intuition is that overlapping modules allow for scenarios where biological components are involved in multiple functions. The workflow is also an *extension* of WGCNA because two additional constraints are considered: networks in the intermediate steps are forced to be scale-free [8] and LASSO regression [9] selects the most relevant modules of responsive genes. The regularized regression technique of LASSO forces the coefficients associated to the less relevant modules to be assigned the value zero [10]; it is particularly useful in scenarios where the number of variables is much larger than the number of samples. This condition is satisfied when the target variables represent the overlapping communities (obtained with HLC) and the samples represent genotype data, which is usually a small set due to the high cost of the RNA sequencing process. Finally, the proposed workflow is also modular, since other module detection and selection techniques could be explored instead of HLC and LASSO.

The approach was showcased with a systematic study on rice (*Oryza sativa*), a food source that is known to be highly sensitive to salt stress [11]. RNA-seq data was accessed from the GEO database [12] (accession number GSE98455). It represents 57, 845 gene expression profiles of shoot tissues measured under control and stress conditions in 92 accessions of the Rice Diversity Panel 1 [13]. A total of 6 modules were detected as relevant in the response to salt stress in rice: 3 modules, each grouping together 3 genes, are associated to shoot K content; 2 modules of 3 genes are associated to shoot biomass; and 1 module of 4 genes is associated to root biomass. These genes are potential targets for experimental validation of salinity tolerance. From the 19 genes, 16 are also identified as deferentially expressed for at least one of the 92 accessions, which re-enforces the labeling of the genes as stress responsive. Moreover, independent recent studies report that 5 of these 19 genes have been identified, through in vivo experimentation, to saline stress. Other genes have GO-annotations related to saline stress, or are reported to have conserved heritability for both control and salt stress conditions. Further studies are needed to elucidate the detailed biological functions of the remaining genes and their role in the mechanisms that respond to salt conditions.

Paper outline The remainder of the paper is organized as follows. The “Preliminaries” section gathers foundations on gene co-expression networks, HLC, and LASSO. The proposed workflow is presented in “The workflow” section, which emphasizes on the logical steps of the data analysis process and the internal structures supporting the approach. The “Case study” section presents an application of the workflow for the identification of rice genes that are sensitive to salt stress. Finally, the “Concluding remarks” section draws some conclusions and future research directions.

## Preliminaries

This section presents preliminaries on networks, the clustering algorithm HLC, and the linear regression technique LASSO.

### Co-expression network

A *network* is an undirected graph $G=(V,E)$ where ${V=\{v_1,v_2,\ldots ,v_{n}\}}$ is a set of *n* *vertices* (or *nodes*) and ${E=\{e_1,e_2,\ldots ,e_q\}}$ is a set of *q* *edges* (or *links*) that connect vertices. In a co-expression network of genes, each node corresponds to a gene and a link indicates a common expression pattern between two genes. The network can be represented by an adjacency matrix $A \in \{0,1\}^{n \times n}$ that is symmetric. A matrix entry in positions $(v_i,v_j)$ and $(v_j,v_i)$ is equal to 1 whenever there is an edge connecting vertices $v_i$ and $v_j$, and equal to 0 otherwise. Co-expression networks are of biological interest because adjacent nodes in the network represent co-expressed genes that are usually controlled by the same transcriptional regulatory pathway, functionally related, or members of the same pathway or metabolic complex [14].

### Hierarchical link clustering

The Hierarchical Link Clustering (HLC) algorithm partitions groups of links (rather than nodes), where each node inherits all memberships of its links and can belong to multiple, overlapping communities [6]. More specifically, HLC evaluates the similarity between links if they share a particular node. Consider a pair of incident links $e_{ik}$ and $e_{jk}$ to node *k*. The similarity between $e_{ik}$ and $e_{jk}$ is defined by the Jaccard index as

1 $$\begin{aligned} S(e_{ik},e_{jk}) = \frac{\vert \ \eta (i) \cap \eta (j) \ \vert }{\vert \ \eta (i) \cup \eta (j) \ \vert }, \end{aligned}$$

where $\eta (v)$ denotes the set containing the node *v* and its neighbors, for any $v \in V$. The algorithm uses single-linkage hierarchical clustering to build a dendrogram where each leaf is a link from the network and branches represent linked communities.

The threshold to cut the dendrogram is defined based on the average density of links inside communities (i.e., partition density). For $G=(V,E)$ and a partition of the links into *c* subsets, the partition density is computed as

2 $$\begin{aligned} D = \frac{2}{\vert E \vert } \sum _c \vert E_c \vert \frac{\vert E_c \vert - \vert V_c \vert + 1 }{(\vert V_c \vert -1)(\vert V_c \vert -2)}. \end{aligned}$$

**Fig. 1:**
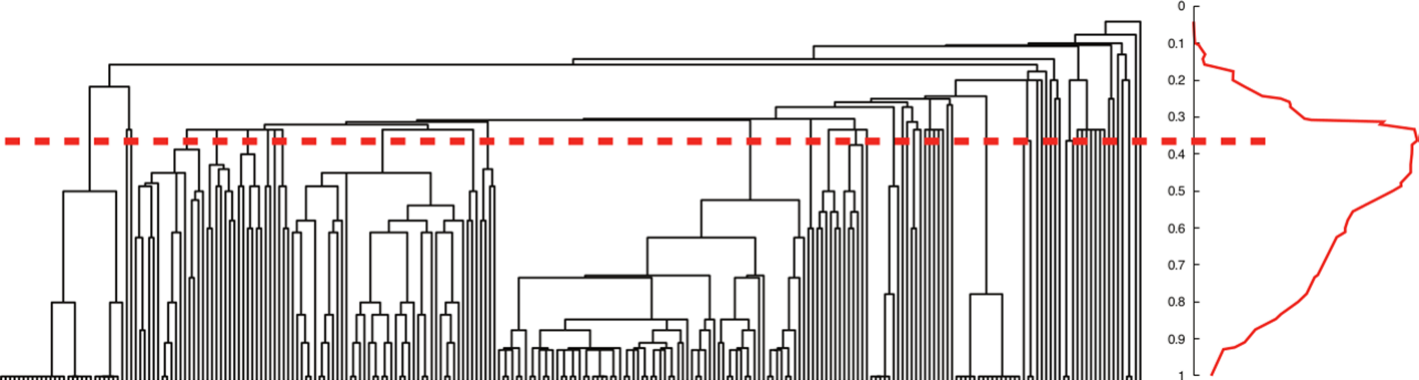
Example of a full link dendrogram (left) and partition density (right), borrowed from [6]

Note that, in most cases, the partition density *D* has a single global maximum along the dendrogram. As depicted in Fig. 1, if the dendrogram is cut at the top, then *D* represents the average link density of a single giant community. If the dendrogram is cut at the bottom, then most communities consist of a single link. In other words, $D = 1$ when every community is a clique and $D = 0$ when each community is a tree. If a community is less dense than a tree (i.e., when the community subgraph has disconnected components), then such a community contributes negatively to *D*, which can take negative values. The minimum density inside a community is $-2/3$, given by one community of two disconnected edges. Since *D* is the average of the intra-community density, there is a lower bound of $-2/3$ for *D*. By computing *D* at each level of the dendrogram, the level that maximizes partition density can be found (nonetheless, meaningful structure could exist above or below the threshold).

The output of the cut is a set of node clusters, where each node can participate in multiple communities.

### Least absolute shrinkage selector operator (LASSO)

LASSO is a regularized linear regression technique. By combining a regression model with a procedure of contraction of some parameters towards 0, LASSO imposes a restriction (or a penalty) on regression coefficients. In other words, LASSO solves the least squares problem with restriction on the $L_1$-norm of the coefficient vector. In particular, the approach is especially useful in scenarios where the number of variables *c* is much greater than the number of samples *m* (i.e., $c \gg m$).

Consider a dataset of *m* samples, consisting each of *c* covariates and a single outcome. Let $y_i$ be the outcome and $x_i := (x_{i1},...,x_{ic})$ be the covariate vector for the *i*-th sample. The objective of LASSO is to solve

3 $$\begin{aligned} \min \left\{ \sum _{i=1}^{m}{\left( y_i-\sum _{j=1}^c{\alpha _j x_{ij}}\right) ^2} \right\} \quad , \quad \text {subject to} \quad \sum _{j=1}^c \left| \alpha _j \right| \le s, \end{aligned}$$

where *s* is the regularization penalty. Equivalently, in the Lagrangian form, LASSO minimizes

4 $$\begin{aligned} \sum _{i=1}^{m}{\left( y_i-\sum _{j=1}^c{\alpha _j x_{ij}}\right) ^2} + \lambda \sum _{j=1}^c \left| \alpha _j \right| , \end{aligned}$$

where $\lambda \ge 0$ is the corresponding Lagrange multiplier. Since the value of the regularization parameter $\lambda$ determines the degree of penalty and the accuracy of the model, cross-validation is used to select the regularization parameter that minimizes the mean-squared error. LASSO is preferred in the proposed workflow because it tends to outperform other methods such as ordinary least squares regression and Ridge [15].

## The workflow

**Fig. 2:**
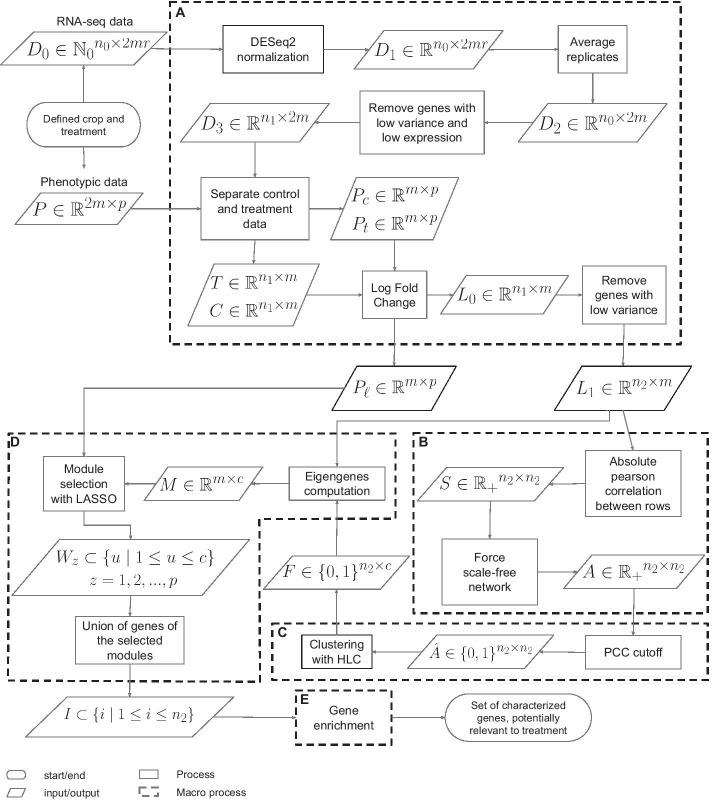
The proposed workflow is broken down into five macro-steps: **a** Data pre-processing, **b** Co-expression network construction, **c** Identification of co-expression modules, **d** Detection of modules association to phenotypic traits, and **e** Gene enrichment

Figure 2 introduces the proposed workflow. It can be broken down into five macro-processes (A)-(E). Compared to WGCNA, the workflow adds the macro-step (D) and generalizes macro-steps (A)-(C).

The input of the workflow includes RNA-seq read counts, representing gene expression levels. More precisely, the workflow uses $n_0$ gene expression profiles measured for *m* different genotypes of *r* biological replicates (under control and treatment conditions). This data is represented as matrix $D_0 \in {{\mathbb {N}}_0}^{n_0 \times 2mr}$. To discover key genes and their interaction with phenotypes related to treatment, the approach also requires a set of *p* phenotypic traits measured for *m* genotypes. The phenotypic data is captured by matrix $P \in {\mathbb {R}}^{2m \times p}$, which contains two phenotypic values per genotype (under control and treatment conditions).

### A. Data pre-processing

The goal of the data pre-processing stage is to build matrices $P_\ell$ and $L_1$ representing, respectively, the changes in phenotypic values and expression levels between control and treatment condition. In other words, $P_\ell$ and $L_1$ are constructed from RNA-seq and phenotypic data found in matrices $D_0$ and *P*.

A normalization process is applied to interpret RNA-seq data and handle possible biases affecting the quantification of results. Here, DESeq2 [16] is used to correct the library size and RNA composition bias. The normalized data is represented as a matrix $D_1 \in {\mathbb {R}}^{n_0 \times 2mr}$, and the biological replicates of each genotype are averaged and represented as a matrix $D_2 \in {\mathbb {R}}^{n_0 \times 2m}$. The genes exhibiting low variance or low expression are removed from $D_2$. Consequently, this stage of the approach reduces the set of genes from a pool of size $n_0$ to a restricted pool of size $n_1 \le n_0$. The control and treatment data is separated into the matrices $C\in {\mathbb {R}}^{n_1 \times m}$ and $T\in {\mathbb {R}}^{n_1 \times m}$, respectively. The matrix entries $c_{ij}$ in *C* and $t_{ij}$ in *T* represent the normalized expression level of gene *i* in accession *j* under control and treatment condition, respectively. Control and treatment data is also separated from phenotypic data *P*, obtaining two matrices $P_c$ and $P_t$ of dimensions $m \times p$.

In the above configuration, the changes in expression levels and phenotypic values between control and treatment conditions are measured in terms of logarithmic ratios. In the case of expression levels, the log ratios are represented in the Log Fold Change matrix $L_0 \in {\mathbb {R}}^{n_1 \times m}$, where $\ell _{ij}=\log _2 (t_{ij}/c_{ij})$. Similarly, the log ratios of the phenotypic data are computed and represented in the $P_\ell \in {\mathbb {R}}^{m \times p}$ matrix.

The final stage of pre-processing is to filter $L_0$ by removing rows (e.g., genes) with low variance in the differential expression patterns, thus obtaining a new matrix $L_1$ of dimensions $n_2 \times m$, with $n_2 \le n_1$.

### B. Construction of the co-expression network

A gene co-expression network connects genes with similar expression patterns across biological conditions. The purpose of this step is to describe how to build the co-expression network *A* from the Log Fold Change matrix $L_1$: the goal is to capture the relationship between genes according to the change in expression levels between the two studied conditions. These co-expression patterns are meaningful for the identification of genes that are not yet associated to treatment response.

The Log Fold Change matrix $L_1$ is used to build the co-expression network following the first two steps of WGCNA [4]. First, the level of concordance between gene differential expression profiles across samples is measured. To this end, as proposed in WGCNA, the absolute value of the Pearson Correlation Coefficient (PCC) is used as the similarity measure between genes, meaning that pairs of nodes with strong negative correlation are considered connected with the same strength as nodes with strong positive correlation [17]. The resulting values are stored in the similarity matrix $S\in \mathbb {R_{+}}^{n_2 \times n_2}$. Second, the matrix *S* is transformed into an adjacency matrix $A \in \mathbb {R_+}^{n_2\times n_2}$ where each entry $a_{ij} = (s_{ij})^\beta$ encodes the connection strength between each pair of genes. In other words, the elements of the adjacency matrix are the similarity values up to the power $\beta > 1$ so that the degree distribution will fit a scale-free network. These networks contain many nodes with very few connections and a small number of hubs with high connections. In a strict scale-free network, the logarithm of *P*(*k*) (i.e., the probability of a node having degree *k*) is approximately inversely proportional to the logarithm of *k* (i.e., the degree of a node). The parameter $\beta$ is chosen to be the smallest value for which the $R^2$ of the linear regression between $log_{10}(p(k))$ and $log_{10}(k)$ is closest to 1 (here, $R^2 > 0.8$).

### C. Identification of co-expression modules

The next step in the workflow is to identify modules of overlapping communities from the co-expression network represented by *A*. The idea is to cluster genes with similar patterns of differential expression change. Membership in these modules may overlap in biological contexts, because modules may be related to specific molecular, cellular, or tissue functions, and the biological components (i.e., genes) may be involved in multiple functions. Unlike WGCNA, the workflow applies the Hierarchical Link Clustering (HLC) algorithm (overviewed in the “Preliminaries” section) to detect overlapping rather than non-overlapping communities.

First, the adjacency matrix *A* is transformed into an unweighted network ${\hat{A}} \in \{0,1\}^{n_2 \times n_2}$. To this end, the PCC cutoff is determined using the approach described in [18]. The number of nodes, edges, and the network density is determined for different PCC cutoffs. In a neighborhood of the optimal PCC cutoff, the number of nodes presents a linear decrease and the density of the network reaches its minimum, while below this value the number of edges rapidly increases. Following this observation, a cutoff is selected such that gene pairs having a correlation score higher than the threshold are considered to have a significant level of co-expression. The entries of *A* become 1 above the cutoff and 0 otherwise. The HLC algorithm organizes the $n_2$ genes of matrix ${\hat{A}}$ into *c* modules, where each gene can belong to zero or multiple modules. This information is represented as an affiliation matrix $F \in \{0,1\}^{n_2 \times c}$, where $f_{iu} = 1$ if node *i* is a member of module *u* (and $f_{iu}=0$, otherwise).

### D. Detection of modules association to phenotypic traits

Each module is represented by an eigengene, which is defined as the first principal component of such module. An eigengene can be seen as an average differential expression profile for each community: it is computed from the Log Fold Change Matrix $L_1$ and the affiliation matrix *F*. Given a module *u*, the affiliation matrix *F* is used to identify the genes belonging to *u*. The corresponding rows of the matrix $L_1$ are selected to compute the first principal component of *u*. Each principal component becomes a column of the matrix $M \in {\mathbb {R}}^{m \times c}$.

These profiles are associated with each phenotypic trait using LASSO as a feature selection mechanism [19]. Therefore, to identify the most relevant modules associated with the phenotypic response to the specific treatment, the eigengenes (i.e., the columns of *M*) act as regressor variables and each phenotypic trait (i.e., each column of $P_\ell$) is used as an outcome variable. LASSO is applied $z \in \{1,2,...,p\}$ times, once for each phenotypic trait. Recall that $y_i$ in Equation 4 is the phenotypic response for the *i*-th sample $(i \in \{1,2,...,m\})$, $x_{ij}$ is the *i*-th value of the eigengene that represents the *j*-th module $(j \in \{1,2,...,c\})$, and the weight $\alpha _j$ represents the importance of the *j*-th module in the phenotypic response. The regularization parameter $\lambda$, tuned with cross-validation, determines the number of modules to be selected. The weights $\alpha$ evolve with each LASSO iteration, by trying to minimize the value of Equation 4, until the desired number of modules with non-zero weight is found. Intuitively, the repetitive use of LASSO in the workflow achieves the goal of neglecting (i.e., reducing to zero) the weights associated to modules with non-essential effects in the phenotypic response and, at the same time, enhancing the weights associated to modules with significant effects.

The output after the repetitive application of LASSO is a set $W_z$ of modules for each phenotypic trait *z*, where $W_z \subseteq \{u \mid 1 \le u \le c\}$ for $z= 1,2,..,p$. A target gene in *I* for downstream analysis is any gene belonging to a selected module; that is, $I = \cup _{z=1}^{p} W_z$, where $I \subseteq \{i \mid 1 \le i \le n_2\}$.

### E. Gene enrichment

This is the final step of the workflow. Its goal is to annotate with additional information the genes identified in previous stages, helping to elucidate their possible behavior and role in the response to the treatment under study.

A crucial step is to identify the differentially expressed genes in the set *I*. That is, to select the genes in *I* that have an absolute value of the Log Fold Change of at least 2 ($|\ell _{ij}|\ge 2$) for at least one sample. This corresponds to genes whose expression level is quadrupled (up or down) from the control to treatment condition; they are the target genes.

Furthermore, functional category enrichment can be carried out by, e.g., searching for gene ontology annotations in databases such as QuickGO [20], UniProt [21], and the Rice Genome Annotation Project [22]. Such annotations can provide evidence of biological implications of the target genes in the treatment-tolerance mechanisms. Furthermore, those databases can be used to identify the protein products of genes, which can be used in turn to provide new insights on how target genes are involved in functional pathways related to treatment. Such analysis includes a review of reported protein-protein interactions in databases such as STRING [23]. The protein interactions include direct (physical) and indirect (functional) associations. They stem from computational prediction, knowledge transfer between organisms, and interactions aggregated from other (primary) databases. The search for unknown interactions would extend the workflow with additional steps.

## Identifying potential saline stress responsive genes in rice

This section presents a case study, applying the approach introduced in “The workflow” section, for identifying genes that respond to saline stress in *Oryza sativa*. The goal of this case study is to discover groups of genes whose differential expression patterns are highly related to phenotypic responses to salt stress. The discovery process is validated with a Fisher’s exact test, thus ensuring that the number of differentially expressed genes (DEG) and of reported genes related to salt stress is statistically significant.

The RNA-seq data was accessed from the GEO database [12] (accession number GSE98455). It corresponds to $n_0={57,845}$ gene expression profiles of shoot tissues measured for control and salt conditions in $m=92$ accessions of the Rice Diversity Panel 1 [13], with $r=2$ biological replicates. A total of $p=3$ phenotypic traits were used: shoot *K* content, and root and shoot biomass. These traits were measured for the same 92 genotypes, under control and treatment conditions, and can be found in the supplementary information for [24].

### A. Data pre-processing

DESeq2 normalization was applied to the raw data and the biological replicates were averaged. Genes exhibiting low variance were identified as those with ratio of upper quantile to lower quantile smaller than 1.5 and were removed from the normalized data. Genes with low expression, corresponding to those having more than $80\%$ samples with values smaller than 10, were also removed. After this filtering process a total of $n_1 = {9,414}$ genes were kept for further analysis.

Genes whose difference between the upper and lower quantiles was greater than 0.25 were removed from the Log Fold Change matrix $L_0$. Therefore, the resulting matrix $L_1$ contained the log ratios of $n_2 = {8,928}$ genes. The logarithmic ratios of the phenotypic data, for the 92 accessions and the 3 traits, were also computed.

### B. Construction of the co-expression network

**Fig. 3:**
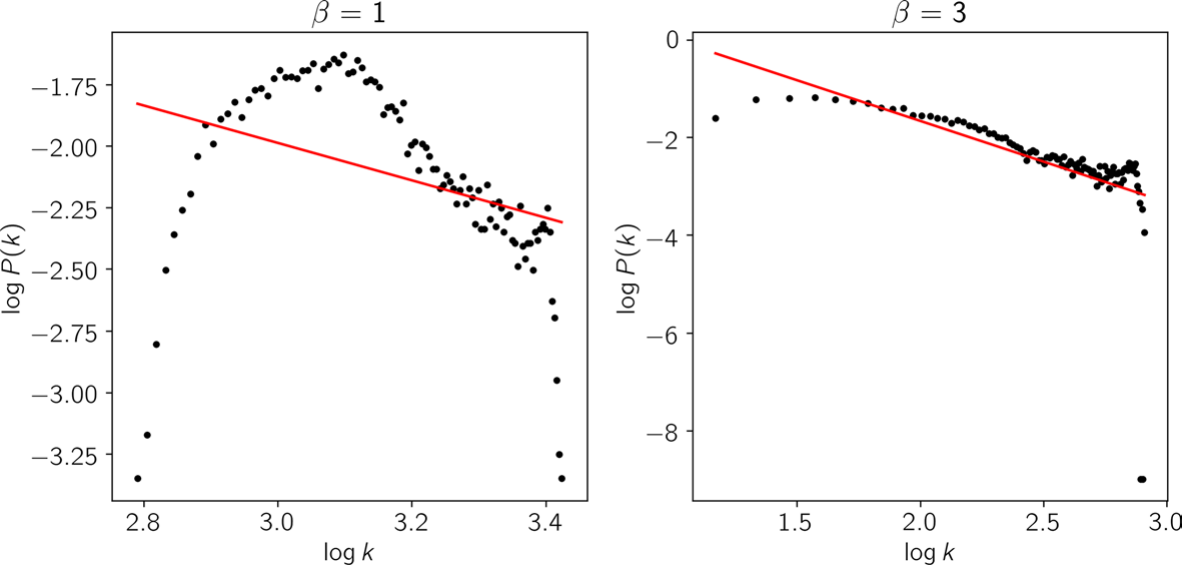
Degree distribution of the network represented by *S* (left) and *A* (right)

The Log Fold Change matrix $L_1$ was used to compute the corresponding similarity matrix. For this network, it was observed that $\beta =3$ is the smallest integer such that $R^2 \ge 0.8$. Figure 3 depicts the degree distribution of the similarity matrix (left) and the degree distribution of the adjacency matrix (right), which is the degree distribution of a scale-free network with $R^2 = 0.8$ and $\beta = 3$.

The resulting adjacency matrix *A* represents a complete graph $G=(V,E)$, with $|V| = {8,928}$ genes ($|E| = {39,850,128}$ edges).

### C. Identification of co-expression modules

The adjacency matrix *A* was transformed into an unweighted network ${\hat{A}}$ applying the approach described in [18]. The cutoff value was set to 0.2, based on the density of the network combined with the decreasing number of nodes and edges with higher PCC values. Hence, only connections above this threshold were kept, while isolated nodes were removed. The resulting adjacency matrix ${\hat{A}}$ consists of 5, 810 connected genes and accounts for 614, 501 edges.

**Fig. 4:**
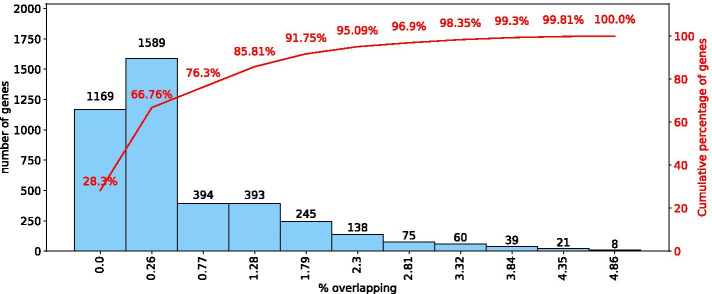
Overlapping percentage of genes after applying HLC

The HLC algorithm distributes 4, 131 genes in $c = {5,143}$ overlapping modules of at least 3 genes each. Figure 4 presents a histogram of the overlapping percentage of these genes, measured as the proportion of modules to which each gene belongs. The first bar of the histogram represents the genes with zero overlap, corresponding to $28\%$ of the total genes; the remaining $72\%$ genes belong to more than one module.

### D. Detection of module association to phenotypic traits

**Fig. 5:**
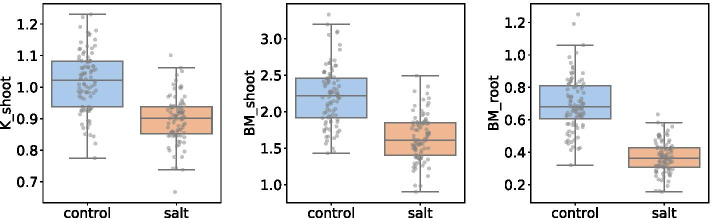
Phenotypic traits distribution under control and salt stress

The phenotypic traits under study are shoot *K* content, and root and shoot biomass. Figure 5 suggests that there are significant differences in the values of these phenotypic traits between stress and control conditions. This supports the working hypothesis that these three variables represent tolerance-associated traits in rice under salt stress.

**Fig. 6:**
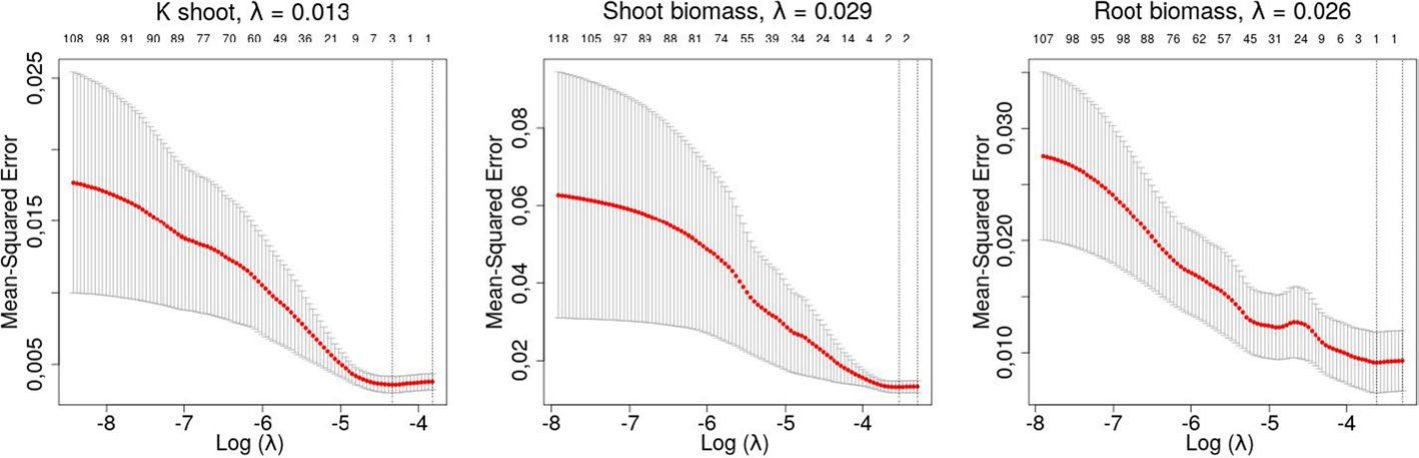
Cross-validation of the LASSO regularization parameter $\lambda$, for each phenotypic trait

By using the affiliation matrix *F* derived from the HLC output and the Log Fold Change matrix $L_1$, a matrix *M* was built by computing the eigengene for each of the $c = {5,143}$ modules. LASSO was applied by using each of the phenotypic traits as the outcome variable, one at a time. As shown in Fig. 6, cross-validation was performed for each phenotypical trait to select the corresponding regularization parameter $\lambda$ minimizing the mean-squared error.

**Fig. 7:**
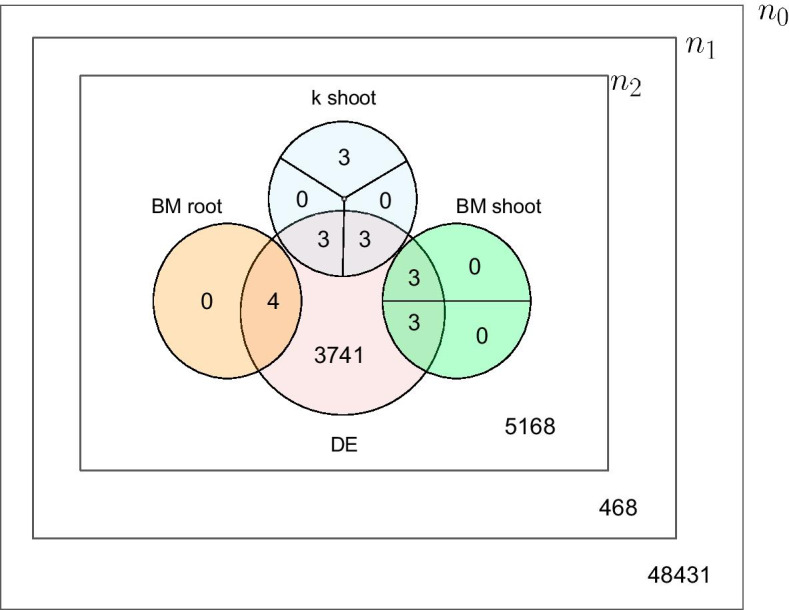
Venn diagram representing the number of genes selected at different stages of the proposed workflow for the case study in rice

Three LASSO models were adjusted by using the corresponding $\lambda$ and phenotypical data with the eigengenes of matrix *M*. As result, 6 modules were detected as relevant in the response to salt stress in rice: 3 modules of 3 genes, each associated with shoot *K* content; 2 modules of 3 genes associated with shoot biomass; and 1 module of 4 genes associated with root biomass. Figure 7 depicts in a Venn diagram how the number of genes selected at different stages evolved.

### E. Gene enrichment

From the 19 genes selected by LASSO, 16 genes ($84\%$) were also identified as differentially expressed ($|\ell _{ij}| \ge 2$) for at least one of the 92 accessions. In general, there were 3, 741 unselected differentially expressed genes and 5, 168 unselected non-differentially expressed ones, for a total of 8, 909 genes. Therefore, differentially expressed genes were significantly more likely to be selected by the workflow, as checked by a Fisher exact test with p-value less than $10^{-3}$.

Figure 7 summarizes how, from the initial $n_0={57,845}$ genes, the proposed workflow identified a reduced set of 19 genes. First, 48, 431 genes were discarded after filtering the normalized expression data $D_2$ and then 486 additional genes were discarded when filtering the Log Fold Change matrix $L_0$. A final set of 19 genes are identified, of which 16 are differentially expressed.

The 19 selected genes were also enriched by contrasting them with findings reported in the literature [25–28], which applied different approaches to study the same RNA-seq dataset GSE98455. In [27], 11 of the 19 selected genes were reported to have conserved heritability for both control and salt stress conditions.

The identifiers for the 19 genes are listed in Table 1. Differentially expressed genes are identified by the mark (*) in column DEG, and those with heritable expression under control and salt stress (as reported in the literature) in column H.

**Table 1:** Selected genes

Phenotypic trait	Module	TU ID	LOC_Os ID	DEG	H
K_shoot	1	13101.t01457	LOC_Os01g16124	*	*
		13101.t01458	LOC_Os01g16130	*	*
		13104.t01366	LOC_Os04g16230	*	
	2	13104.t01068	LOC_Os04g12520	*	*
		13104.t01069	LOC_Os04g12530	*	*
		13104.t01066	LOC_Os04g12499	*	*
	3	13101.t00913	LOC_Os01g10400		
		13102.t03795	LOC_Os02g41820		*
		13103.t00468	LOC_Os03g05870		*
BM_shoot	4	13101.t02836	LOC_Os01g33450	*	*
		13102.t01261	LOC_Os02g14520	*	
		13107.t03589	LOC_Os07g39390	*	
		13112.t00905	LOC_Os12g10280	*	
BM_root	5	13101.t05133	LOC_Os01g58100	*	
		13112.t02444	LOC_Os12g27254	*	
		13112.t03421	LOC_Os12g37260	*	*
	6	13104.t03155	LOC_Os04g35010	*	*
		13108.t03971	LOC_Os08g42310	*	*
		13109.t01501	LOC_Os09g17049	*	

Salinity tolerance comes from genes that limit the rate of salt uptake from the soil and the transport of salt throughout the plant, adjust the ionic and osmotic balance of cells in roots and shoots, and regulate leaf development and the onset of senescence [29]. GO terms related to these characteristics, and therefore relevant to salt stress, were found in this case study to be associated with some selected genes. For example, gene LOC_Os12g37260 is annotated with response to abiotic stimulus and response to stress, and gene LOC_Os12g10280 is annotated with response to extracellular stimulus, channel activity, and transmembrane transport. Genes LOC_Os04g12499, LOC_Os04g12530, and LOC_Os12g10280 are annotated with transporter activity, while gene LOC_Os04g35010 is annotated with multicellular organism development.

**Fig. 8:**
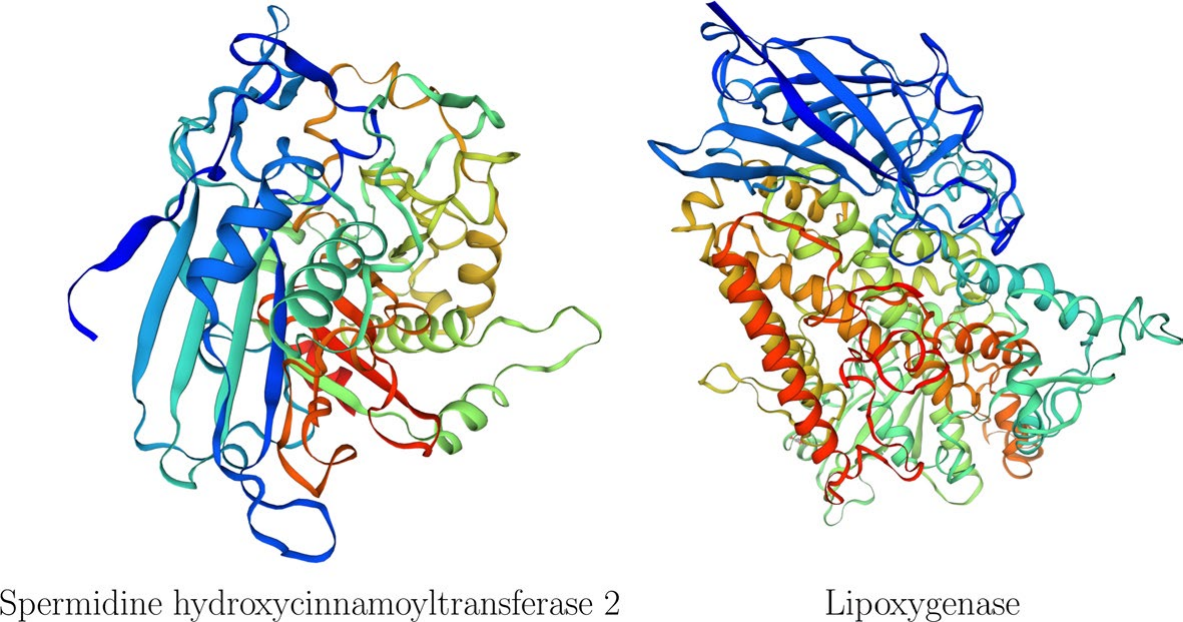
3D protein structure of named genes selected by LASSO, borrowed from [23]

In vivo experiments, reported by independent authors, provide evidence on the relationship with salt stress of 5 genes among the ones selected in the case study ($26\%$). Gene LOC_Os04g12530 is reported as an up-regulated gene in rice plants tolerant to salt stress [30]. Gene LOC_Os12g10280 encodes an aquaporin nodulin 26-like intrinsic membrane (NIP3;5) protein [31]; it has been shown that NIPs play an important role in salt stress responses and in maintaining plant water balance [32]. Gene LOC_Os04g35010 encodes a protein from the bHLH domain, which have been shown to be part of multiple cellular processes, including salt stress signaling pathways [33]. Gene LOC_Os12g27254 encodes spermidine hydroxycinnamoyltransferase 2 (SHT2) protein. This protein contributes to the natural variation of spermidine-based phenolamides in rice cultivars, which is known to promote tolerance to saline stress [34–37]. Gene LOC_Os12g37260 encodes the Lipoxygenase protein, which is known to correlate directly with salt tolerance in rice [38–40]. Note that the STRING database reported a protein-protein interaction of the last two mentioned proteins, namely SHT2 and Lipoxygenase, supporting their membership within the same module, as seen in Table 1. Figure 8 shows the corresponding 3D protein structures of these two proteins. In relation to the 5 genes above-mentioned, there are 387 other genes known to be involved in salt stress (see [30, 41, 42]). Therefore, it can be said that the number of genes selected by the workflow that are related to salt stress is significant, as checked by a Fisher exact test with p-value less than $10^{-2}$.

As a conclusion, the results presented in this section strongly suggest that the proposed workflow, based on identifying overlapping communities in co-expression networks, is capable of detecting stress responsive genes. Further studies are needed to elucidate the detailed biological function of the remaining 14 genes –out of the initial 57, 845 genes– that have not been reported in the literature to be related to salt stress response. This study suggests that they may have the potential to intervene in stress responsive mechanisms to salt conditions in rice.

## Concluding remarks

This manuscript provides a detailed description of a network-based analysis workflow for the discovery of key genes responding to a specific treatment in plants. It links transcriptomic with phenotypic data and identifies overlapping gene modules.

The proposed approach was inspired by the workflow suggested in the WGCNA [4]. Its main steps are the preprocessing of the gene expression data, the construction of a co-expression network, the detection of modules within the network, the relation of modules with external information (e.g., phenotypic data), and the enrichment of the identified key genes with additional information. Both approaches are structured in a modular way, which allows modifying and exploring different techniques in each step of the workflow.

The proposed workflow is designed to integrate expression data measured under two different conditions (namely, control and treatment), unlike the usually co-expression-based approaches working with both conditions independently or considering only a single condition. For this purpose, an approach similar to that proposed in [25] is used, where the control and treatment data are compiled in a single matrix using the Log Fold Change measure. Thus, the input to construct the co-expression network is not the expression data, but instead the changes in the expression levels from one condition to the other, making room for capturing the signal of changes caused by the treatment.

An important feature in the proposed workflow is the module detection technique. The co-expression network is computed, as in WGCNA, until a scale-free network is obtained. In the proposed approach, this network is then used to apply the HLC algorithm, a clustering tool capable of detecting overlapping communities. Several approaches of module detection from gene expression have been proposed and are evaluated in [43]. Most of them focus mainly on disjoint (non-overlapping) communities; the techniques described to deal with overlaps are not clustering, but bi-clustering and decomposition methods. It is well known that communities in real networks, including biological ones, are likely to overlap [44]. Thus, the approach presented in this work can be seen as a generalization of the previous approaches, such as WGCNA, with the potential to deal with genes associated to multiple biological processes.

The workflow proposed in this paper was applied in a case study with rice under salt stress. It identified a group of 19 genes, of which 16 were differentially expressed and 5 have been reported to be related to saline stress response in independent in vivo experiments by other authors [30, 32–35, 39]. Moreover, also 5 of the 19 genes have GO-annotations related to saline stress, and 11 genes are reported to have conserved heritability for both control and salt stress conditions.

As future work, other overlapping module detection and selection techniques should be used, complementing HLC and LASSO, respectively. The combination of these techniques would allow finding target genes for future biological studies that evaluate their potential as genes that respond to salt stress in rice, and other crops and stresses. In vivo laboratory experimentation needs to be conducted to validate the findings of this paper in relation to salinity stress for some of the 19 genes.

Finally, the workflow is presented as a protocol capable of considerably reducing the number of genes detected as relevant in the response to a given stress. Other traditionally used methods for this purpose tend to generate a large list of candidate genes, thus limiting subsequent efforts in experimental validation. In this sense, the proposed workflow can help in reducing such efforts in time and money invested by researchers in the experimental validation of stress-responsive genes.

## Data Availability

The datasets analyzed for the current study are publicly available from different sources. They can be found in the following locations: $\bullet$ RNA-seq data of salt stress in rice is available on the GEO (GSE98455). $\bullet$ Phenotypic data of salt stress in rice is a subset of the supplementary file 1 included in [24]. The data collected, cleaned, and processed from the above sources as used in the case study can be requested to the authors. A workflow implementation is publicly available: $\bullet$ Project name: Condition-specific co-expression network analysis $\bullet$ Project home page: https://github.com/criccio35/workflow_stress $\bullet$ Operating system(s): platform independent. $\bullet$ Programming language: Python 3. $\bullet$ Other requirements: None. $\bullet$ License: GNU GPL v3.

## Abbreviations

GO: Gene ontology
HLC: Hierarchical link clustering
LASSO: Least absolute shrinkage selector operator
PCC: Pearson correlation coefficient
RNA-seq: RNA sequencing
SHT2: Spermidine hydroxycinnamoyltransferase 2
WGCNA: Weighted gene co-expression network analysis
